# Demographics, Cutaneous Manifestations, and Comorbidities Associated with Progressive Cutaneous Sarcoidosis: A Retrospective Cohort Study

**DOI:** 10.3390/medicines10100057

**Published:** 2023-10-12

**Authors:** Jonathan Lai, Erik Almazan, Thomas Le, Matthew T. Taylor, Jihad Alhariri, Shawn G. Kwatra

**Affiliations:** Department of Dermatology, Johns Hopkins University School of Medicine, Baltimore, MD 21205, USA; jlai23@jh.edu (J.L.); tle46@jh.edu (T.L.); mtayl140@jh.edu (M.T.T.); jalhari1@jh.edu (J.A.)

**Keywords:** sarcoid, sarcoidosis, cutaneous lupus pernio, retrospective cohort, treatment resistant, erythema nodosum, racial disparities, comorbidities, autoimmune, demographics

## Abstract

**Background**: Sarcoidosis is a multisystem granulomatous disease with a wide variety of presentations and clinical courses. Cutaneous manifestations and comorbidities associated with sarcoid prognosis remain understudied. **Methods**: An EPIC query was run for patients age 18+ at the Johns Hopkins Hospital with a diagnosis of sarcoidosis of the skin according to the ICD-10-CM code D86.3. Data were obtained from a population-based sample of 240 patients from 2015 to 2020. **Results**: A total of 240 patients were included in the cohort study. The mean (SD) age was 43.76 (11.72) years, and 30% of participants were male; 76.25% of patients identified as black, 19.58% as white, and 4.17% as other. The average age of onset in remissive patients was significantly higher than progressive (47 ± 12 vs. 40 ± 10, *p* = 0.0005); 49% of black patients experienced progressive sarcoid compared to 32.6% of white patients (*p* = 0.028). Progressive disease was associated with the presence of lupus pernio (aOR = 3.29, 95% CI, 1.60–6.77) and at least one autoimmune comorbidity (aOR 6.831, 95% CI 1.819–11.843). **Conclusions**: When controlling for patient demographics, lupus pernio and the presence of at least one autoimmune condition were associated with progressive cutaneous sarcoidosis.

## 1. Introduction

Sarcoidosis is a granulomatous disorder of unknown etiology that often presents with systemic manifestations, most commonly involving the lungs, lymph nodes, and skin [1]. In the United States, sarcoidosis is more prevalent in African Americans and females [1,2,3]. African Americans have a disproportionate burden of disease, with higher rates of cutaneous and systemic involvement that can result in a more chronic disease course [4]. Chronic, progressive sarcoidosis remains a challenge because of its higher somatic, psychosocial, and economic burden associated with increased healthcare utilization [5].

Cutaneous manifestations of sarcoidosis occur in about one-third of patients and can be valuable for visible diagnosis and prognosis. Skin lesions can be described as either specific or non-specific depending on either the presence or absence of noncaseating granulomas on biopsy, respectively [6]. 

Previous studies suggest that, overall, specific skin lesions are associated with a poorer disease prognosis compared to non-specific skin lesions [7]. The non-specific skin lesion erythema nodosum has previously been associated with milder radiological stages, less parenchymal involvement, and better prognosis [7,8,9]. In contrast, the specific skin lesion lupus pernio has been associated with later radiological stages, more parenchymal involvement, and worse prognosis [8,9,10]. However, lupus pernio is more common in black patients, while erythema nodosum is more common in white patients, introducing race as a potential confounder for current prognostic associations [11,12]. To our knowledge, no studies have yet looked at cutaneous presentation and prognosis while controlling for race [7,8,9]. In addition, most studies have looked at the prognostic value of cutaneous manifestations for patient populations in Europe rather than the United States [8,9]. 

There are multiple studies on comorbidities in patients with sarcoidosis, citing higher frequencies of pulmonary, liver, heart, autoimmune disease, and cancer compared to controls [13,14]. Studies of sarcoidosis cohorts also demonstrate a high prevalence of hyperlipidemia, obesity, thyroid disease, diabetes, and hypertension [13,15]. However, no studies to date have looked at how the presence of certain comorbidities relates to sarcoid prognosis. Therefore, the aim of this study was to assess associations of cutaneous presentation and comorbidities with cutaneous sarcoid prognosis while controlling for various demographic factors, including race, sex, age, and smoking status, in a patient population at the Johns Hopkins Hospital. 

## 2. Materials and Methods

A retrospective cohort study was conducted through a review of the electronic medical record of patients who received a diagnosis of cutaneous sarcoid between July 2015 and July 2020 at the Johns Hopkins Hospital. The study was deemed exempt by the Johns Hopkins Institutional Review Board because the data were de-identified. The study was conducted in adherence with the Strengthening the Reporting of Observational Studies in Epidemiology (STROBE) and the Reporting of Studies Conducted Using Observational Routinely Collected Health Data (RECORD) guidelines. 

Our study population included patients with an Internal Classification of Diseases, Tenth Revision, Clinical Modification (ICD-10-CM) code D86.3 for sarcoidosis of the skin. Previous use of this code has demonstrated a diagnostic positive predictive value of 0.94 (95% CI 0.97–0.98) for true sarcoidosis of the skin based on a study in Sweden [16]. The index date was defined by the date of first encounter with a diagnosis of sarcoidosis of the skin. In addition to an ICD-10 code, patients included in this study were 18 years or older at the time of diagnosis and had skin lesions confirmed by biopsy and clinical examination. 

Patient demographics, organ involvement, cutaneous findings, and comorbidities were extracted from the medical record. Age of onset was defined either by the first encounter with a diagnosis of cutaneous sarcoidosis or by patient-provided history if onset was before the earliest documented encounter. Follow-up time was determined by the duration between the most recent documented encounter for cutaneous sarcoidosis and the age of onset. Patients lost to follow-up were excluded from the study. For the six patients who had already died, follow-up time was calculated up to their last documented appointment for sarcoid. Specific skin lesions noted included papular, nodular, subcutaneous, ulcerative, and macular lesions, as well as plaques lupus pernio and Lofgren’s syndrome [6]. Non-specific skin lesions included erythema nodosum, pruritis, and clubbing [6]. Per chart review, Lofgren’s syndrome was diagnosed based on a triad of acute onset erythema nodosum, bilateral hilar/mediastinal lymphadenopathy, and arthritis. Cutaneous manifestations were determined from a physical exam by a dermatologist and diagnosis via biopsy from a pathologist. Comorbidities were determined at the time of diagnosis of cutaneous sarcoidosis. 

Prognosis was determined from a review of follow-up encounter data using previously established criteria [7]. Progressive sarcoidosis was defined by resistance to first-line systemic glucocorticoid therapy and second-line immunomodulators such as methotrexate, finally followed by use of third-line agents such as tumor necrosis factor (TNF)-alpha inhibitors [7,8,17,18]. Examples of the latter include infliximab and adalimumab (see Table S1 for a full list of relevant medications). Other stand-alone criteria included the presence of additional organ involvement after initial diagnosis and/or deterioration of symptoms despite steroid treatment. Attempts to reduce subjective bias regarding clinical symptoms included the use of set descriptors in the chart, such as “well-controlled”, “uncontrolled”, “stable”, and “in remission”. If patients did not meet any of these criteria for progressive sarcoidosis, they were classified as either remissive or stable, based on whether their symptoms completely resolved or remained controlled under constant therapy. 

Data cleaning and analyses were performed using Stata/SE, v. 15.1 (StataCorp LLC, College Station, TX, USA). Categorical variables were compared with chi-squared tests or Fischer’s exact tests. Continuous variables were compared with the Wilcoxon–Mann–Whitney test. Multivariable logistic regression analyses were conducted to assess how the type of cutaneous manifestation and comorbidities predicted progressive disease. Models were adjusted for race, age, sex, and smoking status (non-smoker, previous smoker/current smoker). Models were also adjusted for multiple hypothesis testing with the Benjamini–Hochberg correction. 

## 3. Results

A total of 261 patients were initially identified with a diagnosis of cutaneous sarcoidosis. Eleven patients were excluded because they had inaccessible charts or were lost to follow-up. Ten additional patients were excluded because the cutaneous manifestation type was not reported. In total, 240 patients included in this study had cutaneous sarcoidosis diagnosed by biopsy and clinical findings. 

Of the 240 patients included in the final cohort, 72 (30%) were male, 168 (70%) were female, 183 (76.3%) were black, 47 (19.58%) were white, and 10 (4.17%) were of another race. Overall, 147 (61.3%) of patients were non-smokers, 72 (30%) were previous smokers, and 19 (7.9%) were current smokers. Six (3%) patients were deceased at the time of the study, with three deaths attributable to complications from sarcoidosis. Given the small sample size of other races, only data from black and white patients were analyzed.

Based on progressive systemic involvement, symptom exacerbation, and treatment course, 130 (54.17%) of patients were classified as having either remissive or stable disease, while 107 (44.58%) of patients were classified as having progressive disease. Regarding the age of diagnosis, patients with progressive disease were diagnosed significantly earlier (40 ± 10 years) compared to patients with remissive disease (47 ± 12 years, *p* = 0.0005). Patients with progressive disease also had significantly longer follow-up times (15 ± 11 years) compared to patients with remissive disease (9.4 ± 9.1 years, *p* = 0.0003). Finally, black patients were more likely to have progressive disease (88 [49%]) compared to white patients (15 [33%], *p* = 0.028) (Table 1). Neither sex nor smoking status was significantly associated with disease prognosis.

Black patients were diagnosed significantly earlier than white patients (41.8 ± 11.5 years vs. 49.8 ± 10 years, *p* = 0.001). In addition, black patients had significantly longer follow-up time compared to white patients (14.2 ± 13 years vs. 8.3 ± 7.9 years, *p* = 0.004) (Table 2). Among the different categories of cutaneous lesions, no differences were noted in either age of onset (*p* = 0.83) or follow-up time (*p* = 0.35) for sarcoidosis-specific and sarcoidosis non-specific lesions. In addition, no differences were noted regarding age of onset or follow-up time for any particular lesion compared to the rest of the cohort. 

There were no significant differences in the frequency of sarcoidosis-specific (*p* = 0.16) and sarcoidosis non-specific lesions (*p* = 0.72) between blacks and whites. When looking at lesion subcategories, lupus pernio disproportionately affected blacks compared to the rest of the cohort (44 [91.7%] vs. 139 [75.9%], *p* = 0.022). In contrast, the frequency of occurrence of erythema nodosum among black patients was not significantly different from the rest of the cohort (14 [82.4%] vs. 169 [79%], *p* = 0.51) (Table 3). 

After adjusting for age of onset, race, sex, and smoking status, disease progression was not associated with the presence of sarcoidosis-specific cutaneous manifestations when compared to non-specific sarcoidosis cutaneous manifestations (102 [50.5%] vs. 3 [18.8%]; adjusted odds ratio (aOR) 3.32, 95% CI 0.87–12.59). However, we demonstrate that lupus pernio was the only skin lesion associated with progressive disease (35 [72.9%] vs. 72 [37.5%]; (aOR) 3.29, 95% CI 1.60–6.77) (Table 4).

We then stratified by race and found that the association of lupus pernio with progressive disease persisted in the cohort of only black patients ((aOR) 4.07, 95% CI 1.83–9.02), and only white patients ((aOR) 17.5, 95% CI 1.55–196.3) (Table 5).

We did not find a significant association between any individual comorbidity and progressive sarcoidosis. However, we found that the presence of any autoimmune comorbidity was associated with progressive disease ((aOR) 6.831, 95% CI 1.819–11.843) (Table 6). Any autoimmune comorbidity included inflammatory arthritis, Crohn’s disease, sicca syndrome, systemic lupus erythematosus, autoimmune thyroid conditions, and other autoimmune conditions.

## 4. Discussion

In this retrospective cohort study, we identified associations between progressive cutaneous sarcoidosis and black race, early age of onset, lupus pernio, and the presence of a concomitant autoimmune comorbidity at diagnosis. Consistent with previous studies, we demonstrated that cutaneous sarcoidosis is a disease that disproportionately affects African Americans [4,11,19]. Contrary to several other studies, we did not observe associations between progressive cutaneous sarcoidosis and specific skin lesions aside from lupus pernio, nor did we observe associations between remissive/stable cutaneous sarcoidosis and the presence of erythema nodosum [7,8]. In addition, we did not find an association between progressive sarcoidosis and sex. Types of comorbidities associated with progressive sarcoidosis have been understudied in the literature, and to our knowledge, our study is the first to do so. 

Our study adds to the growing body of literature that cutaneous sarcoid disproportionately affects African Americans and is also more likely to be progressive and treatment-resistant. Similar to a recent cohort study in the United States, we demonstrated that African American women are most frequently and severely affected by sarcoidosis [7]. Other studies have also noted that African Americans with sarcoidosis experience higher levels of sarcoid-related mortality, greater granuloma density, increased treatment resistance, and more frequent development of new organ findings [4,20,21,22]. 

Also consistent with previous studies, we demonstrated that African Americans tend to present with sarcoid at a younger age than Caucasians [19,23]. The earlier age of onset of sarcoidosis in African Americans is consistent with our finding that sarcoid diagnosed earlier is also associated with progressive disease. Given that sarcoidosis is more likely to present chronically in African Americans, it is not surprising that follow-up times are also longer in this demographic. However, we also demonstrated that a younger age of diagnosis is independently associated with progressive sarcoidosis. Age of onset has not previously been studied in the adult sarcoid population, but early onset sarcoidosis in pediatric populations has been associated with a poorer long-term prognosis [24]. More studies describing age-related prognostic implications of sarcoidosis in adult populations are required.

In addition to race and age of onset, we identified an association between lupus pernio and progressive sarcoidosis. This finding has been described in retrospective cohort studies in Spain and Turkey but, to our knowledge, has not yet been observed in a patient population in the United States [7,9]. Similar to previous studies, lupus pernio disproportionately affected African Americans in our cohort [11,12]. Unlike previous studies, we demonstrate that after controlling for patient demographics such as age, gender, and race, lupus pernio is still significantly associated with a worse prognosis. Given the persistence of this association after stratifying for race, this indicates that lupus pernio is independently associated with progressive disease. This finding illustrates that a clinical diagnosis of lupus pernio in non-black populations is still associated with a poorer prognosis and should be approached and treated as such. 

Except for lupus pernio, other cutaneous manifestations, including both sarcoidosis-specific and sarcoidosis non-specific, were not associated with progressive disease. Our multivariate analysis did not validate previous studies that the presence of erythema nodosum is associated with remissive/stable disease [6,7,25]. Given previous literature supporting an association of erythema nodosum with good prognosis, we can provide two explanations for our findings. The first is that previous associations of erythema nodosum with good prognosis could have been confounded by race or age at diagnosis. To our knowledge, these studies used chi-square and student t-tests to compare outcomes, did not control for potential confounding variables, and did not adjust for multiple comparisons. After controlling for these variables, we demonstrated that the association disappears. The second explanation is that our study lacked the power to detect this association. While erythema nodosum was the most frequent skin lesion in a previous cohort study (20.5%), it was relatively uncommon in our cohort (7.1%), perhaps because our population was based in a tertiary care center that overrepresents patients with more progressive disease. In addition, previous studies were conducted in Europe and Asia, which tend to have less severe radiological patterns and more frequent skin involvement [7,8,9,26]. These geographical differences may also be indirectly responsible for a difference in the frequency of erythema nodosum. A future study with an increased sample size could address this issue. 

Sex is a well-known mediator of sarcoidosis, with previous studies demonstrating associations with certain symptoms, relapse rates, and overall mortality [27]. In our study, we did not observe an association between sex and disease progression. However, we demonstrated an overall higher female prevalence in both the progressive and remissive sarcoid cohorts, consistent with prior studies [28]. When we examined the association of sex with various parameters, we found a greater percentage of white males and black females in the total (*p* = 0.003) and progressive (*p* = 0.001) cohorts. We also found that females had a significantly longer follow-up time in the total (*p* = 0.0123) and progressive (*p* = 0.006) cohorts (Table S2). Interestingly, our findings are consistent with previous studies suggesting that white males may have higher rates of sarcoid relapse compared to other races and sex groups [29]. We did not corroborate previous findings suggesting a higher rate of late-onset sarcoidosis in females, nor did we find evidence to support a higher rate of cutaneous sarcoid involvement in females [8,29]. In comparison to Judson et al., our study examined patients with cutaneous sarcoid and did not exclusively use pulmonary function tests as a surrogate for disease progression, highlighting differences in patient type and methodology as potential reasons for sex discrepancies [29]. 

Finally, our study is the first to assess associations between concomitant comorbidities at sarcoid diagnosis and sarcoid prognosis. Despite our understanding of the etiology of sarcoid remaining unknown, there is a general consensus of an underlying immune-mediated process driving sarcoid pathogenesis [30]. Such processes include granuloma formation via the interplay between macrophages and T cells in response to a culprit antigen and loss of tolerance to self-antigens on the T-cell level [31]. B-cell-mediated processes may also contribute, given a recent study demonstrating the resolution of sarcoidosis in some patients after rituximab treatment [32]. The coexistence of sarcoidosis with other autoimmune diseases has suggested the possibility of a common pathogenesis and genetic predisposition. These include the HLA-DR3 genotype in Sjogren’s and the HLA-B27 genotype in spondylarthritis [33]. Autoimmune disorders have been shown to be more common in sarcoidosis patients compared to the general population [34,35]. A retrospective study in Taiwan demonstrated a higher risk of autoimmune thyroid disease, Sjogren’s syndrome, and ankylosing spondylitis in patients with sarcoidosis [31]. Another study in Europe demonstrated that compared to the general population, sarcoidosis patients had a two-fold higher chance of having at least one associated autoimmune condition [31,32]. Our findings on the prognostic implications of a concomitant autoimmune condition on sarcoid raise the possibility of a subpopulation of “inflammatory” sarcoid patients who may be more resistant to first-line treatment in part due to overall immune dysregulation. 

This study has several limitations. As with all observational studies, we cannot conclude any causal relationships from our findings. Retrospective medical records introduce provider variability regarding documentation of cutaneous manifestations, symptoms, and comorbidities. In addition, because charts were obtained at a tertiary medical center, our results are not generalizable to the entire US, with a potential selection bias for patients with a more chronic disease course. As mentioned earlier, a small sample size could have an impact on our negative findings regarding erythema nodosum. Similarly, a future study looking at associations between progressive disease and lupus pernio should aim to have a higher sample size of white patients diagnosed with lupus pernio because only five patients fulfilled that category in our study. As such, our findings can serve as a preliminary framework for a future study looking at a more robust real-world data set. Finally, some aspects of disease outcome were prone to subjective interpretation, especially symptom progression, on chart abstraction. An attempt to mitigate this bias was made by using specific phrases such as “worsening”, “progressive”, and “resistant”. 

## 5. Conclusions

In conclusion, our study reconfirms gender and racial differences in the incidence of cutaneous sarcoidosis, highlights racial differences in severity, and suggests that age at diagnosis, lupus pernio, and presence of at least one autoimmune comorbidity are associated with a poorer cutaneous sarcoid prognosis. The differences we found in the prognostic utility of cutaneous presentation compared to previous studies suggest geographical, institutional, and epidemiological variability, especially for erythema nodosum. Our findings on cutaneous manifestations of sarcoidosis emphasize that regardless of age, sex, or race, lupus pernio continues to present as a sign of progressive sarcoidosis that may warrant the use of second and third-line therapies for treatment. Finally, we further the notion of an overlap between sarcoidosis and autoimmune conditions and suggest that patients with both are more likely to experience progressive disease and treatment resistance, possibly due to overlapping immunologic mechanisms. 

## Figures and Tables

**Table 1:** Demographic data of stable vs. progressive patients.

	Remission/Stable (*n* = 133)	Progressive (*n* = 107)	*p*-Value ^1^
Age (years), mean (SD)	47 (12)	40 (10)	<0.0001
Follow-up time (years), mean (SD)	9.4 (9.1)	14.8 (11.2)	<0.0001
Sex, *n* (%)			
Male	39 (30)	33 (31)	0.84
Female	94 (70)	72 (69)	
Race/Ethnicity, *n*(%)			
Non-Hispanic White	32 (24)	15 (14)	0.028
Black	93 (70)	80 (84)	
Other	8 (6)	2 (2)	
Smoking History, *n* (%)			
Never	81 (61)	66 (62)	0.887
Former/Current	51 (38)	40 (37)	

^1^ α = 0.05, *p* values adjusted using the Benjamini–Hochberg method.

**Table 2:** Demographic data of black and white patients.

	Mean (SD) Age of Onset (Years)	*p*-Value ^1^	Mean (SD) Follow-Up Time (Years)	*p*-Value ^1^
Race				
Black	41.82 (11.46)	<0.0001	14.19 (13.02)	0.0011
White	49.79 (10.02)		8.25 (7.89)	
Cutaneous				
Specific	43.39 (11.55)	0.19	13.7 (14.7)	0.35
Non-specific	45.86 (12.2)		11.5 (8.7)	
Papular	43.64 (11.78)	0.99	12.7 (15.8)	0.60
Nodular	43.35 (11.13)	0.76	12.9 (10.2)	0.65
Subcutaneous	44.82 (11.45)	0.47	12.6 (10.9)	0.69
Macular	44.31 (11.95)	0.69	12.3 (10.8)	0.59
Plaque	44.99 (11.67)	0.20	13.5 (15.9)	0.95
Lupus pernio	42.39 (11.58)	0.41	14.6 (11.1)	0.51
Ulcerative	38 (8.77)	0.17	12.8 (13.2)	0.90
Erythema Nodosum	44.59 (11.02)	0.74	11.5 (8.9)	0.58
Pruritis	46.3 (12.79)	0.36	11.6 (7.9)	0.54
Clubbing	39 (1)	0.58	14.3 (5.9)	0.93
Lofgren’s	52	–	2.2	–

^1^ α = 0.05, *p* values adjusted using the Benjamini–Hochberg method.

**Table 3:** Distribution of cutaneous lesions in sarcoid patients.

Cutaneous Manifestations	Number (%) of Patients	Number (%) Black Patients	*p*-Value ^1^
Categories			
Specific	224 (93.3)	170 (75.9)	0.16
Non-specific	43 (17.92)	33 (84.62)	0.72
Lesion types			
Papular	82 (34.17)	64 (83.12)	0.49
Nodular	88 (36.67)	67 (77.91)	0.73
Subcutaneous	44 (18.33)	29 (69.05)	0.42
Macular	42 (17.5)	34 (80.95)	0.52
Plaque	84 (35)	72 (88.89)	0.014
Lupus pernio	49 (21)	44 (88)	0.022
Ulcerative	8 (3.33)	7 (87.5)	0.58
Erythema nodosum	17 (7.08)	14 (82.35)	0.58
Pruritis	21 (8.75)	14 (82.35)	1
Clubbing	2 (0.083)	2 (100)	1
Lofgren’s	1 (0.047)	1 (100)	

^1^ α = 0.05, *p* values adjusted using the Benjamini–Hochberg method.

**Table 4:** Multivariate logistic regression for the type of cutaneous involvement of sarcoid and odds of progressive disease.

Cutaneous Involvement (Yes/No)	Remission/Stable (*n* = 133, %)	Progressive (*n* = 107, %)	Adjusted Odds Ratio ^1^	95% Confidence Intervals	*p*-Value ^1,2^
Specific, *n* (%)					
No	13 (10)	3 (3)	3.32	0.87–12.59	0.16
Yes	116 (90)	102 (97)			
Non-Specific, *n* (%)					
No	100 (78)	91 (87)	0.57	0.27–1.16	0.12
Yes	29 (22)	14 (13)			
Papular	47 (36)	32 (30)	0.72	0.40–1.29	1
Nodular	47 (36)	40 (38)	1.02	0.58–1.79	1
Subcutaneous	23 (18)	21 (20)	1.30	0.65–2.63	1
Macular	18 (14)	22 (21)	1.78	0.86–3.70	1
Plaque	47 (36)	35 (33)	0.87	0.48–1.56	1
Lupus pernio	13 (9)	37 (36)	3.29	1.60–6.77	0.01
Ulcerative	3 (2)	4 (4)	1.10	0.22–5.35	1
Erythema nodosum	10 (8)	7 (6)	0.88	0.31–2.52	1
Pruritic	14 (11)	7 (6)	0.67	0.24–1.83	1
Clubbing	1 (1)	1 (1)	1.01	0.06–16.88	0.99
Lofgren’s	1 (1)	0 (0)	1^2^	-	–

^1^ Controlled for age at diagnosis, race, sex, and smoking status (never, previous, current); ^2^ α = 0.05, *p* values adjusted using the Benjamini–Hochberg method.

**Table 5:** Associations of lupus pernio and progressive sarcoidosis stratified by race.

Cutaneous and Racial Category	Remission/Stable (*n* = 133, %)	Progressive (*n* = 107, %)	Adjusted Odds Ratio ^1^	95% Confidence Intervals	*p*-Value ^2^
Black, lupus pernio	11 (8)	33 (31)	4.07	1.83–9.02	0.002
Black, no lupus pernio	77 (58)	62 (58)			
White, lupus pernio	1 (1)	4 (4)	17.5	1.55–196.3	0.021
White, no lupus pernio	44 (33)	8 (7)			

^1^ Controlled for age at diagnosis, sex, and smoking status (never, previous, current); ^2^ α = 0.05, *p* values adjusted using the Benjamini–Hochberg method.

**Table 6:** Multivariate logistic regression for comorbidities associated with progressive sarcoidosis.

Comorbidities	N (%)	Adjusted Odds Ratio ^1^	95% Confidence Intervals	*p*-Value ^1,2^
Medical allergy	159 (66%)	2.825	1.254–4.397	0.0557
Food allergy	57 (24%)	1.784	0.768–2.801	0.601
Asthma	48 (20%)	1.884	0.666–3.102	0.782
Inflammatory arthritis	23 (9.6%)	7.764	1.034–14.493	0.2442
Osteoarthritis	7 (3%)	1100.5	0–2201	0.99
Crohn’s disease	2 (0.8%)	12.135	0.087–24.183	0.99
Sicca syndrome	4 (1.7%)	35.931	0.429–71.433	0.601
Osteoporosis	6 (2.5%)	784	0–1568	0.99
Chronic kidney disease	17 (7.1%)	5.475	0.066–10.884	0.99
Sleep apnea	41 (17%)	6.985	0.917–13.053	0.2948
Gastroesophageal reflux disease	65 (27%)	1.408	0.482–2.333	0.99
Chronic heart failure	22 (9.2%)	9.353	0.49–18.217	0.601
Depression	33 (13.8%)	2.158	0.354–3.962	0.99
Anxiety	25 (10.4%)	2.719	0.381–5.058	0.99
Systemic lupus erythematosus	5 (2.1%)	56.579	0.148–113.01	0.811
Type II diabetes mellitus	70 (29.3%)	1.419	0.481–2.358	0.99
Chronic obstructive pulmonary disease	12 (5%)	7.196	0.303–14.09	0.842
Dyslipidemia	51 (21.3%)	2.629	0.683–4.575	0.601
Autoimmune thyroid disease	31 (13%)	9.932	1.665–18.198	0.0557
Malignancy	20 (8.3%)	2.025	0.125–3.924	0.99
Other autoimmune condition	8 (3.33%)	4.171	0.057–8.284	0.99
Any autoimmune condition	56 (23.3%)	6.831	1.819–11.843	0.0286

^1^ Controlled for age at diagnosis, race, sex, and smoking status (never, previous, current). ^2^ α = 0.05, *p* values adjusted using the Benjamini–Hochberg method.

## Data Availability

J. Lai and S. Kwatra had full access to the database population. Supplemental information, including the study protocol, raw data, and statistical code can be accessed by emailing jlai23@jh.edu.

## Supplementary Materials

The following supporting information can be downloaded at https://www.mdpi.com/article/10.3390/medicines10100057/s1, Table S1: Associations of lupus pernio and progressive sarcoidosis stratified by race. Table S2: Sociodemographics and Comorbidities Stratified by Sex.
