# Supplementary material for: Demographics, Cutaneous Manifestations, and Comorbidities Associated with Progressive Cutaneous Sarcoidosis: A Retrospective Cohort Study
Source: Medicines (Basel). 2023 Oct 12;10(10):57. doi: 10.3390/medicines10100057 (PMC10608652; doi:10.3390/medicines10100057)
Supplement: Supplementary file 1 [file medicines-10-00057-s001.zip › medicines-2535708-supplementary.pdf]

Supplementary Materials

Table S1. Associations of Lupus Pernio and Progressive Sarcoidosis Stratified by Race.

Cutaneous and Racial Category	Remission/Stable (*n* = 133, %)	Progressive (*n* = 107, %)	Adjusted Odds Ratio [1]	95% Confidence Intervals	*p*-Value [2]
Black, Lupus Pernio	11 (8)	33 (31)	4.07	1.83–9.02	**0.002**
Black No Lupus Pernio	77 (58)	62 (58)			
White, Lupus Pernio	1 (1)	4 (4)	17.5	1.55–196.3	**0.021**
White, No Lupus Pernio	44 (33)	8 (7)			

[1] Controlled for age at diagnosis, sex, and smoking status (never, previous, current). [2] $\alpha = 0.05$, *p* values adjusted using the Benjamini-Hochberg method.

Table S2. Sociodemographics and Comorbidities Stratified by Sex.

	Full Cohort			Progressive Cohort		
Total (N)	72<br>Male (%)	168<br>Female (%)	*p* [1]	33<br>Male	74<br>Female	*p* [1]
Black	45 (67)	138 (84)		22 (69)	68 (93)	
White	22 (33)	25 (15)	0.003	10 (31)	5 (7)	0.001
Age (years), mean (SD)	43.5 (11.2)	43.7 (12)	0.8648	41.1 (8.9)	39.3 (9.9)	0.3968
Follow-up time (years), mean (SD)	9.9 (8.1)	13.6 (10.9)	0.0123	11.3 (8.6)	17.4 (11.4)	0.006
Specific	67 (93)	157 (93)	0.656	33 (100)	74 (100)	0.519
Non-specific	12 (17)	31 (18)	0.803	4 (12)	10 (14)	0.936
Papular	22 (31)	60 (36)	0.698	10 (30)	23 (31)	0.936
Nodular	24(33)	64 (38)	0.698	11 (33)	29 (39)	0.936
Subcutaneous	16 (22)	28 (17)	0.656	7 (21)	14 (19)	0.936
Macular	13 (18)	29 (17)	0.822	10 (30)	13 (18)	0.519
Plaque	27 (38)	57 (34)	0.735	14 (42)	21 (29)	0.519
Lupus Pernio	13 (18)	35 (21)	0.735	8 (24)	21 (28)	0.936
Ulcerative	1 (1)	7 (4)	0.656	0 (0)	4 (5)	0.519
Erythema Nodosum	3 (4)	14 (8.3)	0.656	2 (6)	5 (7)	0.936
Pruritis	9 (13)	12 (7)	0.656	3 (9)	4 (5)	0.936
Clubbing	0 (0)	2 (1)	0.656	0 (0)	1 (1)	0.936
Lofgren's	1 (1)	0 (0)	0.656	0	0	
Medical Allergy	41 (57)	118 (70)	0.356	20 (61)	59 (80)	0.479
Food Allergy	13 (18)	44 (26)	0.481	6 (18)	22 (30)	0.479
Asthma	19 (26)	29 (17)	0.385	10 (30)	14 (19)	0.479
Inflammatory Arthritis	4 (6)	19 (11)	0.481	2 (6)	12 (16)	0.479
Osteoarthritis	1 (1)	6 (4)	0.667	0 (0)	3 (4)	0.479
Crohn's Disease	0 (0)	2 (1)	0.667	0 (0)	1 (1)	0.736
Sicca Syndrome	1 (1)	3 (2)	0.909	1 (3)	2 (3)	0.952
Osteoporosis	1 (1)	5 (30)	0.811	1 (3)	2 (3)	0.479
Chronic Kidney Disease	6 (8)	11 (7)	0.877	4 (12)	5 (7)	0.602
Sleep Apnea	13 (18)	28 (17)	0.909	6 (18)	15 (20)	0.927
Gastroesophageal Reflux Disease	14 (19)	51 (30)	0.356	6 (18)	22 (30)	0.479
Chronic Heart Failure	6 (8)	16 (10)	0.909	4 (12)	8 (11)	0.927
Depression	10 (14)	23 (14)	1	7 (21)	9 (12)	0.479
Anxiety	7 (10)	18 (11)	0.909	3 (9)	7 (9)	0.952
Systemic Lupus Erythematosus	1 (1)	4 (2)	0.877	0 (0)	3 (4)	0.479
Type II Diabetes Mellitus	15 (21)	55 (33)	0.356	8 (24)	21 (28)	0.836
Chronic Obstructive Pulmonary Disease	2 (3)	10 (6)	0.667	1 (3)	7 (9)	0.479
Dyslipidemia	18 (25)	33 (20)	0.667	6 (18)	16 (22)	0.836

Autoimmune Thyroid Disease	5 (7)	26 (15)	0.356	5 (15)	13 (18)	0.479
Malignancy	6 (8)	14 (8)	1	2 (6)	8 (11)	0.479
Other Autoimmune Condition	3 (4)	5 (3)	0.667	2 (6)	3 (4)	0.836
Any Autoimmune Condition	11 (15)	45 (27)	0.356	6 (18)	25 (34)	0.479

[1] $\alpha = 0.05$, p values adjusted using the Benjamini-Hochberg method.
